# 6,7-Diphenyl-5-thia-7-aza­spiro­[2.6]nonan-8-one

**DOI:** 10.1107/S1600536813027979

**Published:** 2013-10-19

**Authors:** Hemant P. Yennawar, Lee J. Silverberg

**Affiliations:** a Department of Chemistry, Pennsylvania State University, University Park, PA 16802, USA; b Penn State University, Schuylkill Campus, 200 University Drive, Schuylkill Haven, PA 17972, USA

## Abstract

The asymmetric unit of the title compound, C_19_H_19_NOS, contains two independent mol­ecules (*A* and *B*), in both of which the 1,3-thia­zepan-4-one ring adopts a chair-type conformation. The dihedral angles between the two phenyl rings are 65.28 (8) and 60.31 (9)° for mol­ecules *A* and *B*, respectively. In the crystal, mol­ecules are linked by weak C—H⋯O inter­actions, resulting in a three-dimensional network.

## Related literature

For amide bond formation using 2,4,6-tripropyl-1,3,5,2,4,6-trioxatri­phospho­rinane–2,4,6-trioxide (T3P), see: Dunetz *et al.* (2011 ▶). For preparation of various heterocycles using imines and T3P, see: Unsworth *et al.* (2013 ▶). For omapatrilat, see: Graul *et al.* (1999 ▶); Robl *et al.* (1997 ▶); Tabrizchi (2001 ▶).
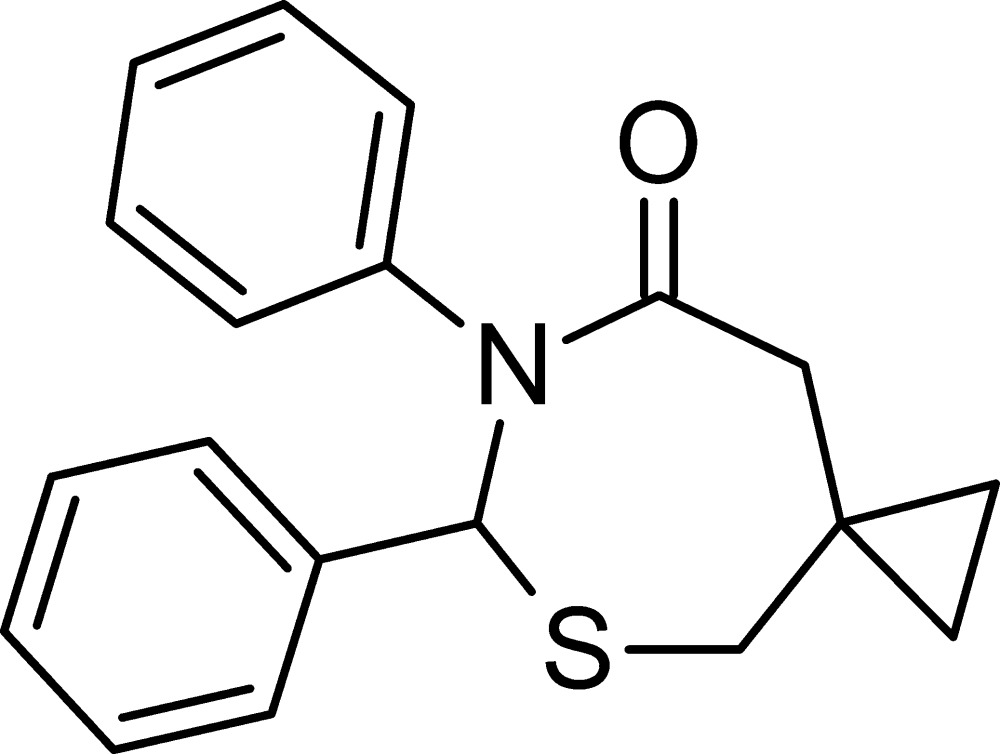


## Experimental

### 

#### Crystal data

C_19_H_19_NOS
*M* *_r_* = 309.41
Triclinic, 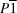

*a* = 9.9954 (18) Å
*b* = 10.695 (2) Å
*c* = 16.397 (3) Å
α = 79.764 (3)°
β = 83.659 (3)°
γ = 73.048 (3)°
*V* = 1646.8 (5) Å^3^
*Z* = 4
Mo *K*α radiation
μ = 0.20 mm^−1^
*T* = 298 K
0.29 × 0.28 × 0.12 mm

#### Data collection

Bruker SMART APEX CCD diffractometer
Absorption correction: multi-scan (*SADABS*; Bruker, 2001 ▶) *T*_min_ = 0.559, *T*_max_ = 1.000
14502 measured reflections
7089 independent reflections
5751 reflections with *I* > 2σ(*I*)
*R*_int_ = 0.017

#### Refinement

*R*[*F* ^2^ > 2σ(*F* ^2^)] = 0.047
*wR*(*F* ^2^) = 0.134
*S* = 1.02
7089 reflections
397 parameters
H-atom parameters constrained
Δρ_max_ = 0.22 e Å^−3^
Δρ_min_ = −0.19 e Å^−3^

### 

Data collection: *APEX2* (Bruker, 2001 ▶); cell refinement: *SAINT* (Bruker, 2001 ▶); data reduction: *SAINT*; program(s) used to solve structure: *SHELXS97* (Sheldrick, 2008 ▶); program(s) used to refine structure: *SHELXL97* (Sheldrick, 2008 ▶); molecular graphics: *ORTEP-3 for Windows* (Farrugia, 2012 ▶); software used to prepare material for publication: *SHELXL97*.

## Supplementary Material

Crystal structure: contains datablock(s) I. DOI: 10.1107/S1600536813027979/lx2289sup1.cif

Structure factors: contains datablock(s) I. DOI: 10.1107/S1600536813027979/lx2289Isup2.hkl

Supplementary material file. DOI: 10.1107/S1600536813027979/lx2289Isup3.mol

Supplementary material file. DOI: 10.1107/S1600536813027979/lx2289Isup4.cml

Additional supplementary materials:  crystallographic information; 3D view; checkCIF report

## Figures and Tables

**Table 1:** Hydrogen-bond geometry (Å, °)

*D*—H⋯*A*	*D*—H	H⋯*A*	*D*⋯*A*	*D*—H⋯*A*
C18—H18*A*⋯O2^i^	0.97	2.60	3.364 (2)	136
C38—H38*A*⋯O1	0.97	2.53	3.384 (2)	146

Symmetry code: (i) 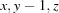.

**Fig. 1.**
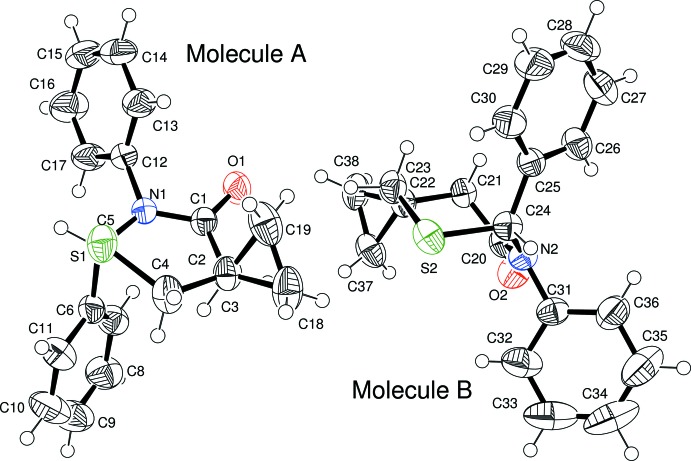
ORTEP view of the title comound. Thermal ellipsoids are drawn at 50% probability.

**Fig. 2.**
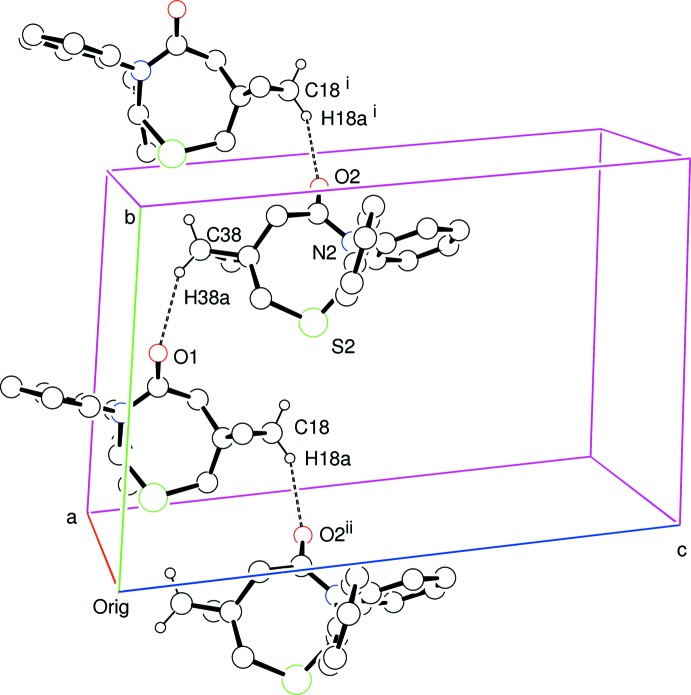
Crystal packing. C–H···O interactions are shown as dashed lines. [Symmetry codes: (i) x, y+1, z; (ii) x, y-1, z.]

## supplementary crystallographic information

### 1. Comment

The seven-membered 1,3-thiazepan-4-one ring system is of biological interest, as exemplified by the investigational compound omapatrilat (Graul *et al.*, 1999; Robl *et al.*, 1997; Tabrizchi, 2001). As part of our studies of cyclic 1,3-thiaza-4-one compounds, we report the synthesis and structure of the novel title compound. The title molecule was synthesized by condensation of *N*-[phenylmethylidene]aniline with [1-(sulfanylmethyl)cyclopropyl]acetic acid in the presence of 2,4,6-tripropyl-1,3,5,2,4,6-trioxatriphosphorinane-2,4,6-trioxide (T3P) and pyridine (Dunetz *et al.*, 2011; Unsworth *et al.*, 2013). We report here the crystal structure of the title compound which crystallizes with two independent molecules, A & B, in the asymmetric unit. In the title compound (Fig. 1), the 1,3-thiazepan-4-one ring adopts a chair type conformation. The dihedral angles formed by the two benzene rings are 65.28 (8)° for molecule A and 60.31 (9)° for molecule B, respectively. In the crystal packing (Fig. 2), molecules are connected by weak C–H···O interactions (Table 1), resulting in a three-dimensional network.

### 2. Experimental

A two-necked 25 ml pear flask was oven-dried, cooled under N_2_, and charged with a stir bar and *N*-[phenylmethylidene]aniline (1.087 g, 6 mmol). Tetrahydrofuran (2.3 mL) was added, the solid dissolved, and the solution was stirred. Pyridine (1.95 ml, 24 mmol) was added and then [1-(sulfanylmethyl)cyclopropyl] acetic acid (0.877 g, 6 mmol) was added. Finally, 2,4,6-tripropyl-1,3,5,2,4,6-trioxatriphosphorinane-2,4,6-trioxide in 2-methyltetrahydrofuran (50 weight percent, 7.1 ml, 12 mmol) was added. The reaction was stirred at room temperature for 20 h, then poured into a separatory funnel with dichloromethane. The mixture was washed with saturated sodium bicarbonate. The aqueous solution was then extracted twice with dichloromethane. The organics were combined and washed twice with saturated sodium bicarbonate, and once each with water and saturated sodium chloride. The organic was dried over sodium sulfate, concentrated *in vacuo* and chromatographed on 29 g flash silica gel, eluting with mixtures of ethyl acetate and hexanes. The product eluted with 30-50% EtOAc/hexanes and was concentrated *in vacuo* to a white solid (0.4594 g). Recrystallization from ethanol gave white crystals (0.2366 g, 12.7%). m.p.: 418-420 K. Crystals for X-Ray Crystallography were grown by slow evaporation from ethanol.

### 3. Refinement

The C-bound H atoms were geometrically placed, with C—H = 0.93–0.97 Å, and refined as riding, with *U*_iso_(H) = 1.2*U*_eq_(C)

### Crystal data

**Table:**

C_19_H_19_NOS	*Z* = 4
*M**_r_* = 309.41	*F*(000) = 656
Triclinic, *P*1	*D*_x_ = 1.248 Mg m^−^^3^
Hall symbol: -P 1	Melting point = 418–420 K
*a* = 9.9954 (18) Å	Mo *K*α radiation, λ = 0.71073 Å
*b* = 10.695 (2) Å	Cell parameters from 5499 reflections
*c* = 16.397 (3) Å	θ = 2.4–27.9°
α = 79.764 (3)°	µ = 0.20 mm^−^^1^
β = 83.659 (3)°	*T* = 298 K
γ = 73.048 (3)°	Block, colorless
*V* = 1646.8 (5) Å^3^	0.29 × 0.28 × 0.12 mm

### Data collection

**Table:**

Bruker SMART APEX CCD diffractometer	7089 independent reflections
Radiation source: fine-focus sealed tube	5751 reflections with *I* > 2σ(*I*)
Parallel,graphite monochromator	*R*_int_ = 0.017
Detector resolution: 8.34 pixels mm^-1^	θ_max_ = 27.0°, θ_min_ = 2.0°
φ and ω scans	*h* = −11→12
Absorption correction: multi-scan (*SADABS*; Bruker, 2001)	*k* = −13→13
*T*_min_ = 0.559, *T*_max_ = 1	*l* = −20→20
14502 measured reflections	

### Refinement

**Table:**

Refinement on *F*^2^	Primary atom site location: structure-invariant direct methods
Least-squares matrix: full	Secondary atom site location: difference Fourier map
*R*[*F*^2^ > 2σ(*F*^2^)] = 0.047	Hydrogen site location: difference Fourier map
*wR*(*F*^2^) = 0.134	H-atom parameters constrained
*S* = 1.02	*w* = 1/[σ^2^(*F*_o_^2^) + (0.0761*P*)^2^ + 0.240*P*] where *P* = (*F*_o_^2^ + 2*F*_c_^2^)/3
7089 reflections	(Δ/σ)_max_ < 0.001
397 parameters	Δρ_max_ = 0.22 e Å^−^^3^
0 restraints	Δρ_min_ = −0.19 e Å^−^^3^

### Special details

**Table:**

Experimental. ^1^H NMR (CDCl_3_): 7.547-7.241 (10 H), 6.161 (s, 1 H), 3.120-3.094 (bd, 1H), 2.730-2.701 (bd, 1H), 2.524 (bs, 2H), 0.888 (bp, 1H), 0.763-0.609 (m, 3H).
Geometry. All e.s.d.'s (except the e.s.d. in the dihedral angle between two l.s. planes) are estimated using the full covariance matrix. The cell e.s.d.'s are taken into account individually in the estimation of e.s.d.'s in distances, angles and torsion angles, correlations between e.s.d.'s in cell parameters are only used when they are defined by crystal symmetry. An approximate (isotropic) treatment of cell e.s.d.'s is used for estimating e.s.d.'s involving l.s. planes.
Refinement. Refinement of *F*^2^ against ALL reflections. The weighted *R*-factor *wR* and goodness of fit *S* are based on *F*^2^, conventional *R*-factors *R* are based on *F*, with *F* set to zero for negative *F*^2^. The threshold expression of *F*^2^ > σ(*F*^2^) is used only for calculating *R*-factors(gt) *etc*. and is not relevant to the choice of reflections for refinement. *R*-factors based on *F*^2^ are statistically about twice as large as those based on *F*, and *R*- factors based on ALL data will be even larger.

### Fractional atomic coordinates and isotropic or equivalent isotropic displacement parameters (Å^2^)

**Table:**

	*x*	*y*	*z*	*U*_iso_*/*U*_eq_	
C1	0.85001 (16)	0.37226 (15)	0.11160 (9)	0.0395 (3)	
C2	0.91058 (17)	0.31239 (15)	0.19446 (9)	0.0412 (3)	
H2A	0.8945	0.3818	0.2283	0.049*	
H2B	1.0111	0.2762	0.1858	0.049*	
C3	0.84951 (18)	0.20409 (16)	0.24162 (10)	0.0460 (4)	
C4	0.9200 (2)	0.06671 (16)	0.22199 (11)	0.0532 (4)	
H4A	0.8826	0.0042	0.2613	0.064*	
H4B	1.0193	0.0459	0.2297	0.064*	
C5	0.97945 (16)	0.16374 (15)	0.05328 (9)	0.0416 (3)	
H5	0.9842	0.1423	−0.0028	0.050*	
C6	1.13156 (17)	0.14856 (16)	0.06834 (10)	0.0445 (4)	
C7	1.18570 (19)	0.25627 (19)	0.05273 (11)	0.0529 (4)	
H7	1.1274	0.3401	0.0354	0.063*	
C8	1.3261 (2)	0.2407 (2)	0.06263 (14)	0.0688 (5)	
H8	1.3608	0.3141	0.0530	0.083*	
C9	1.4136 (2)	0.1172 (3)	0.08663 (16)	0.0824 (7)	
H9	1.5077	0.1065	0.0934	0.099*	
C10	1.3612 (2)	0.0091 (3)	0.10063 (17)	0.0831 (7)	
H10	1.4208	−0.0748	0.1162	0.100*	
C11	1.2211 (2)	0.0236 (2)	0.09179 (13)	0.0629 (5)	
H11	1.1870	−0.0502	0.1015	0.075*	
C12	0.81582 (16)	0.35078 (15)	−0.02817 (9)	0.0420 (3)	
C13	0.67364 (19)	0.3669 (2)	−0.02791 (12)	0.0556 (4)	
H13	0.6234	0.3463	0.0213	0.067*	
C14	0.6062 (2)	0.4137 (2)	−0.10098 (14)	0.0681 (6)	
H14	0.5103	0.4252	−0.1008	0.082*	
C15	0.6806 (2)	0.4433 (2)	−0.17398 (13)	0.0685 (6)	
H15	0.6350	0.4760	−0.2230	0.082*	
C16	0.8219 (2)	0.4245 (3)	−0.17412 (12)	0.0746 (6)	
H16	0.8725	0.4425	−0.2237	0.090*	
C17	0.89034 (19)	0.3790 (2)	−0.10144 (11)	0.0604 (5)	
H17	0.9863	0.3675	−0.1020	0.073*	
C18	0.7963 (2)	0.2142 (2)	0.33049 (12)	0.0688 (6)	
H18A	0.8083	0.1328	0.3695	0.083*	
H18B	0.8035	0.2894	0.3535	0.083*	
C19	0.6963 (2)	0.2399 (2)	0.26482 (14)	0.0671 (6)	
H19A	0.6423	0.3306	0.2481	0.081*	
H19B	0.6472	0.1742	0.2641	0.081*	
C20	0.63401 (16)	0.86157 (15)	0.38230 (10)	0.0409 (3)	
C21	0.57843 (17)	0.87198 (15)	0.29878 (9)	0.0425 (3)	
H21A	0.5910	0.9514	0.2637	0.051*	
H21B	0.4787	0.8804	0.3061	0.051*	
C22	0.65069 (17)	0.75300 (16)	0.25537 (10)	0.0431 (4)	
C23	0.58698 (19)	0.63898 (17)	0.27661 (11)	0.0497 (4)	
H23A	0.6275	0.5769	0.2381	0.060*	
H23B	0.4873	0.6721	0.2689	0.060*	
C24	0.51975 (16)	0.68075 (15)	0.44372 (10)	0.0411 (3)	
H24	0.5180	0.6330	0.5005	0.049*	
C25	0.36604 (16)	0.74436 (16)	0.42803 (9)	0.0421 (3)	
C26	0.30000 (18)	0.87250 (18)	0.44207 (11)	0.0531 (4)	
H26	0.3517	0.9230	0.4576	0.064*	
C27	0.1571 (2)	0.9260 (2)	0.43309 (13)	0.0671 (5)	
H27	0.1140	1.0126	0.4419	0.081*	
C28	0.0790 (2)	0.8517 (3)	0.41122 (14)	0.0724 (6)	
H28	−0.0167	0.8875	0.4054	0.087*	
C29	0.1442 (2)	0.7237 (3)	0.39810 (14)	0.0725 (6)	
H29	0.0917	0.6728	0.3838	0.087*	
C30	0.28607 (19)	0.6703 (2)	0.40589 (12)	0.0568 (5)	
H30	0.3287	0.5840	0.3963	0.068*	
C31	0.67344 (18)	0.75204 (17)	0.52198 (10)	0.0470 (4)	
C32	0.8144 (2)	0.6878 (2)	0.52163 (15)	0.0738 (6)	
H32	0.8629	0.6598	0.4734	0.089*	
C33	0.8830 (3)	0.6655 (3)	0.5937 (2)	0.1034 (10)	
H33	0.9781	0.6213	0.5944	0.124*	
C34	0.8105 (4)	0.7087 (3)	0.66459 (19)	0.1085 (11)	
H34	0.8575	0.6955	0.7126	0.130*	
C35	0.6711 (3)	0.7703 (3)	0.66487 (15)	0.0974 (9)	
H35	0.6225	0.7971	0.7134	0.117*	
C36	0.6012 (2)	0.7933 (2)	0.59327 (12)	0.0684 (5)	
H36	0.5058	0.8364	0.5932	0.082*	
C37	0.80546 (19)	0.72194 (19)	0.23689 (12)	0.0572 (5)	
H37A	0.8538	0.7791	0.2534	0.069*	
H37B	0.8585	0.6295	0.2411	0.069*	
C38	0.7103 (2)	0.7778 (2)	0.16753 (11)	0.0596 (5)	
H38A	0.7053	0.7191	0.1298	0.072*	
H38B	0.7006	0.8688	0.1421	0.072*	
N1	0.88793 (13)	0.30028 (12)	0.04720 (8)	0.0398 (3)	
N2	0.60091 (13)	0.77434 (13)	0.44779 (8)	0.0406 (3)	
O1	0.76816 (14)	0.48252 (11)	0.10230 (8)	0.0553 (3)	
O2	0.70900 (14)	0.92934 (12)	0.39130 (8)	0.0564 (3)	
S1	0.89821 (5)	0.04462 (4)	0.11782 (3)	0.05310 (14)	
S2	0.61188 (5)	0.55158 (4)	0.38153 (3)	0.05175 (14)	

### Atomic displacement parameters (Å^2^)

**Table:**

	*U*^11^	*U*^22^	*U*^33^	*U*^12^	*U*^13^	*U*^23^
C1	0.0417 (8)	0.0372 (8)	0.0412 (8)	−0.0133 (6)	−0.0005 (6)	−0.0074 (6)
C2	0.0484 (9)	0.0394 (8)	0.0380 (8)	−0.0135 (7)	−0.0009 (7)	−0.0104 (6)
C3	0.0539 (10)	0.0412 (8)	0.0419 (8)	−0.0143 (7)	0.0049 (7)	−0.0067 (7)
C4	0.0671 (12)	0.0396 (8)	0.0484 (9)	−0.0129 (8)	0.0057 (8)	−0.0035 (7)
C5	0.0443 (9)	0.0386 (8)	0.0405 (8)	−0.0067 (7)	−0.0010 (7)	−0.0116 (6)
C6	0.0427 (9)	0.0484 (9)	0.0381 (8)	−0.0056 (7)	0.0009 (7)	−0.0093 (7)
C7	0.0458 (9)	0.0575 (10)	0.0513 (10)	−0.0121 (8)	0.0016 (8)	−0.0042 (8)
C8	0.0521 (11)	0.0857 (15)	0.0699 (13)	−0.0261 (11)	0.0023 (10)	−0.0077 (11)
C9	0.0423 (11)	0.109 (2)	0.0902 (17)	−0.0129 (12)	−0.0071 (11)	−0.0121 (14)
C10	0.0531 (12)	0.0779 (15)	0.1000 (18)	0.0089 (11)	−0.0135 (12)	−0.0060 (13)
C11	0.0537 (11)	0.0524 (10)	0.0732 (13)	−0.0014 (9)	−0.0056 (9)	−0.0066 (9)
C12	0.0397 (8)	0.0455 (8)	0.0402 (8)	−0.0090 (7)	−0.0046 (6)	−0.0087 (7)
C13	0.0430 (9)	0.0745 (12)	0.0529 (10)	−0.0214 (9)	−0.0018 (8)	−0.0109 (9)
C14	0.0445 (10)	0.0924 (15)	0.0718 (13)	−0.0186 (10)	−0.0175 (9)	−0.0166 (11)
C15	0.0672 (13)	0.0852 (15)	0.0533 (11)	−0.0169 (11)	−0.0242 (10)	−0.0062 (10)
C16	0.0642 (13)	0.1120 (18)	0.0427 (10)	−0.0242 (13)	−0.0038 (9)	0.0008 (11)
C17	0.0408 (9)	0.0904 (14)	0.0452 (10)	−0.0141 (9)	−0.0019 (7)	−0.0049 (9)
C18	0.0964 (16)	0.0564 (11)	0.0489 (10)	−0.0207 (11)	0.0187 (11)	−0.0109 (9)
C19	0.0631 (12)	0.0542 (11)	0.0835 (15)	−0.0214 (9)	0.0233 (11)	−0.0176 (10)
C20	0.0396 (8)	0.0381 (8)	0.0452 (8)	−0.0127 (6)	0.0048 (7)	−0.0085 (6)
C21	0.0460 (9)	0.0389 (8)	0.0412 (8)	−0.0128 (7)	0.0034 (7)	−0.0045 (6)
C22	0.0428 (9)	0.0442 (8)	0.0436 (8)	−0.0143 (7)	0.0055 (7)	−0.0112 (7)
C23	0.0496 (10)	0.0506 (9)	0.0547 (10)	−0.0197 (8)	0.0072 (8)	−0.0198 (8)
C24	0.0401 (8)	0.0423 (8)	0.0424 (8)	−0.0181 (7)	0.0015 (6)	−0.0020 (6)
C25	0.0373 (8)	0.0523 (9)	0.0372 (8)	−0.0174 (7)	0.0038 (6)	−0.0039 (7)
C26	0.0460 (10)	0.0578 (10)	0.0552 (10)	−0.0166 (8)	0.0067 (8)	−0.0102 (8)
C27	0.0503 (11)	0.0664 (12)	0.0701 (13)	−0.0030 (10)	0.0111 (10)	−0.0039 (10)
C28	0.0372 (10)	0.1025 (18)	0.0692 (13)	−0.0149 (11)	−0.0012 (9)	−0.0005 (12)
C29	0.0471 (11)	0.1038 (18)	0.0773 (14)	−0.0349 (12)	−0.0018 (10)	−0.0197 (13)
C30	0.0472 (10)	0.0675 (12)	0.0627 (11)	−0.0259 (9)	0.0024 (8)	−0.0150 (9)
C31	0.0441 (9)	0.0521 (9)	0.0480 (9)	−0.0211 (7)	−0.0066 (7)	−0.0008 (7)
C32	0.0497 (11)	0.0854 (15)	0.0829 (15)	−0.0173 (11)	−0.0134 (11)	−0.0006 (12)
C33	0.0674 (16)	0.119 (2)	0.122 (3)	−0.0301 (16)	−0.0475 (17)	0.016 (2)
C34	0.126 (3)	0.135 (3)	0.0827 (19)	−0.067 (2)	−0.0608 (19)	0.0192 (18)
C35	0.119 (2)	0.132 (2)	0.0551 (13)	−0.052 (2)	−0.0197 (14)	−0.0131 (14)
C36	0.0685 (13)	0.0908 (15)	0.0495 (11)	−0.0253 (12)	−0.0055 (9)	−0.0134 (10)
C37	0.0448 (10)	0.0526 (10)	0.0742 (13)	−0.0167 (8)	0.0136 (9)	−0.0151 (9)
C38	0.0691 (12)	0.0647 (11)	0.0480 (10)	−0.0261 (10)	0.0150 (9)	−0.0152 (8)
N1	0.0399 (7)	0.0400 (7)	0.0371 (6)	−0.0048 (5)	−0.0042 (5)	−0.0092 (5)
N2	0.0396 (7)	0.0452 (7)	0.0403 (7)	−0.0194 (6)	−0.0011 (5)	−0.0029 (5)
O1	0.0651 (8)	0.0390 (6)	0.0549 (7)	0.0005 (6)	−0.0076 (6)	−0.0120 (5)
O2	0.0652 (8)	0.0551 (7)	0.0601 (7)	−0.0360 (6)	−0.0013 (6)	−0.0068 (6)
S1	0.0614 (3)	0.0428 (2)	0.0607 (3)	−0.0205 (2)	0.0047 (2)	−0.01759 (19)
S2	0.0507 (3)	0.0371 (2)	0.0660 (3)	−0.01365 (18)	0.0064 (2)	−0.00759 (19)

### Geometric parameters (Å, º)

**Table:**

C1—C2	1.506 (2)	C20—C21	1.508 (2)
C1—N1	1.3718 (18)	C20—N2	1.3706 (19)
C1—O1	1.2183 (19)	C20—O2	1.2199 (18)
C2—H2A	0.9700	C21—H21A	0.9700
C2—H2B	0.9700	C21—H21B	0.9700
C2—C3	1.517 (2)	C21—C22	1.523 (2)
C3—C4	1.508 (2)	C22—C23	1.506 (2)
C3—C18	1.506 (2)	C22—C37	1.493 (2)
C3—C19	1.489 (3)	C22—C38	1.505 (2)
C4—H4A	0.9700	C23—H23A	0.9700
C4—H4B	0.9700	C23—H23B	0.9700
C4—S1	1.8100 (18)	C23—S2	1.8118 (19)
C5—H5	0.9800	C24—H24	0.9800
C5—C6	1.524 (2)	C24—C25	1.518 (2)
C5—N1	1.4726 (19)	C24—N2	1.4744 (19)
C5—S1	1.8267 (17)	C24—S2	1.8297 (16)
C6—C7	1.383 (2)	C25—C26	1.385 (2)
C6—C11	1.390 (2)	C25—C30	1.391 (2)
C7—H7	0.9300	C26—H26	0.9300
C7—C8	1.388 (3)	C26—C27	1.389 (3)
C8—H8	0.9300	C27—H27	0.9300
C8—C9	1.372 (3)	C27—C28	1.376 (3)
C9—H9	0.9300	C28—H28	0.9300
C9—C10	1.377 (4)	C28—C29	1.378 (3)
C10—H10	0.9300	C29—H29	0.9300
C10—C11	1.384 (3)	C29—C30	1.377 (3)
C11—H11	0.9300	C30—H30	0.9300
C12—C13	1.380 (2)	C31—C32	1.376 (3)
C12—C17	1.376 (2)	C31—C36	1.376 (3)
C12—N1	1.4391 (19)	C31—N2	1.433 (2)
C13—H13	0.9300	C32—H32	0.9300
C13—C14	1.381 (3)	C32—C33	1.380 (3)
C14—H14	0.9300	C33—H33	0.9300
C14—C15	1.375 (3)	C33—C34	1.379 (4)
C15—H15	0.9300	C34—H34	0.9300
C15—C16	1.367 (3)	C34—C35	1.356 (4)
C16—H16	0.9300	C35—H35	0.9300
C16—C17	1.380 (3)	C35—C36	1.380 (3)
C17—H17	0.9300	C36—H36	0.9300
C18—H18A	0.9700	C37—H37A	0.9700
C18—H18B	0.9700	C37—H37B	0.9700
C18—C19	1.485 (3)	C37—C38	1.491 (3)
C19—H19A	0.9700	C38—H38A	0.9700
C19—H19B	0.9700	C38—H38B	0.9700
			
N1—C1—C2	118.89 (13)	C20—C21—H21B	109.0
O1—C1—C2	120.36 (13)	C20—C21—C22	112.84 (13)
O1—C1—N1	120.74 (14)	H21A—C21—H21B	107.8
C1—C2—H2A	108.8	C22—C21—H21A	109.0
C1—C2—H2B	108.8	C22—C21—H21B	109.0
C1—C2—C3	113.86 (13)	C23—C22—C21	115.21 (13)
H2A—C2—H2B	107.7	C37—C22—C21	118.26 (14)
C3—C2—H2A	108.8	C37—C22—C23	117.80 (14)
C3—C2—H2B	108.8	C37—C22—C38	59.63 (12)
C4—C3—C2	115.73 (14)	C38—C22—C21	118.20 (14)
C18—C3—C2	117.56 (14)	C38—C22—C23	116.52 (14)
C18—C3—C4	116.33 (15)	C22—C23—H23A	108.7
C19—C3—C2	117.76 (15)	C22—C23—H23B	108.7
C19—C3—C4	118.35 (15)	C22—C23—S2	114.33 (12)
C19—C3—C18	59.44 (13)	H23A—C23—H23B	107.6
C3—C4—H4A	108.7	S2—C23—H23A	108.7
C3—C4—H4B	108.7	S2—C23—H23B	108.7
C3—C4—S1	114.30 (13)	C25—C24—H24	103.9
H4A—C4—H4B	107.6	C25—C24—S2	115.68 (11)
S1—C4—H4A	108.7	N2—C24—H24	103.9
S1—C4—H4B	108.7	N2—C24—C25	114.89 (13)
C6—C5—H5	103.9	N2—C24—S2	112.78 (10)
C6—C5—S1	116.07 (11)	S2—C24—H24	103.9
N1—C5—H5	103.9	C26—C25—C24	121.14 (14)
N1—C5—C6	114.66 (13)	C26—C25—C30	118.65 (16)
N1—C5—S1	112.51 (11)	C30—C25—C24	119.99 (15)
S1—C5—H5	103.9	C25—C26—H26	119.8
C7—C6—C5	121.04 (15)	C25—C26—C27	120.44 (18)
C7—C6—C11	118.82 (17)	C27—C26—H26	119.8
C11—C6—C5	119.94 (16)	C26—C27—H27	119.8
C6—C7—H7	119.6	C28—C27—C26	120.4 (2)
C6—C7—C8	120.74 (18)	C28—C27—H27	119.8
C8—C7—H7	119.6	C27—C28—H28	120.4
C7—C8—H8	119.9	C27—C28—C29	119.28 (19)
C9—C8—C7	120.1 (2)	C29—C28—H28	120.4
C9—C8—H8	119.9	C28—C29—H29	119.6
C8—C9—H9	120.2	C30—C29—C28	120.8 (2)
C8—C9—C10	119.5 (2)	C30—C29—H29	119.6
C10—C9—H9	120.2	C25—C30—H30	119.8
C9—C10—H10	119.6	C29—C30—C25	120.46 (19)
C9—C10—C11	120.9 (2)	C29—C30—H30	119.8
C11—C10—H10	119.6	C32—C31—N2	119.67 (17)
C6—C11—H11	120.0	C36—C31—C32	120.55 (19)
C10—C11—C6	119.9 (2)	C36—C31—N2	119.77 (16)
C10—C11—H11	120.0	C31—C32—H32	120.4
C13—C12—N1	120.51 (15)	C31—C32—C33	119.2 (3)
C17—C12—C13	119.90 (16)	C33—C32—H32	120.4
C17—C12—N1	119.56 (15)	C32—C33—H33	120.0
C12—C13—H13	120.1	C34—C33—C32	119.9 (3)
C12—C13—C14	119.84 (17)	C34—C33—H33	120.0
C14—C13—H13	120.1	C33—C34—H34	119.7
C13—C14—H14	119.9	C35—C34—C33	120.5 (2)
C15—C14—C13	120.17 (18)	C35—C34—H34	119.7
C15—C14—H14	119.9	C34—C35—H35	120.0
C14—C15—H15	120.2	C34—C35—C36	120.1 (3)
C16—C15—C14	119.70 (18)	C36—C35—H35	120.0
C16—C15—H15	120.2	C31—C36—C35	119.6 (2)
C15—C16—H16	119.6	C31—C36—H36	120.2
C15—C16—C17	120.72 (19)	C35—C36—H36	120.2
C17—C16—H16	119.6	C22—C37—H37A	117.7
C12—C17—C16	119.66 (18)	C22—C37—H37B	117.7
C12—C17—H17	120.2	H37A—C37—H37B	114.8
C16—C17—H17	120.2	C38—C37—C22	60.61 (12)
C3—C18—H18A	117.8	C38—C37—H37A	117.7
C3—C18—H18B	117.8	C38—C37—H37B	117.7
H18A—C18—H18B	114.9	C22—C38—H38A	117.8
C19—C18—C3	59.69 (13)	C22—C38—H38B	117.8
C19—C18—H18A	117.8	C37—C38—C22	59.76 (12)
C19—C18—H18B	117.8	C37—C38—H38A	117.8
C3—C19—H19A	117.7	C37—C38—H38B	117.8
C3—C19—H19B	117.7	H38A—C38—H38B	114.9
C18—C19—C3	60.87 (13)	C1—N1—C5	124.94 (13)
C18—C19—H19A	117.7	C1—N1—C12	118.35 (13)
C18—C19—H19B	117.7	C12—N1—C5	115.82 (11)
H19A—C19—H19B	114.8	C20—N2—C24	125.52 (13)
N2—C20—C21	119.50 (13)	C20—N2—C31	117.58 (13)
O2—C20—C21	120.25 (14)	C31—N2—C24	115.62 (12)
O2—C20—N2	120.24 (15)	C4—S1—C5	102.63 (8)
C20—C21—H21A	109.0	C23—S2—C24	102.03 (8)
			
C1—C2—C3—C4	87.12 (18)	C25—C24—N2—C20	−68.64 (19)
C1—C2—C3—C18	−129.09 (17)	C25—C24—N2—C31	124.65 (15)
C1—C2—C3—C19	−61.01 (19)	C25—C24—S2—C23	57.87 (12)
C2—C1—N1—C5	3.3 (2)	C25—C26—C27—C28	0.9 (3)
C2—C1—N1—C12	171.94 (13)	C26—C25—C30—C29	0.0 (3)
C2—C3—C4—S1	−67.20 (18)	C26—C27—C28—C29	−0.2 (3)
C2—C3—C18—C19	107.58 (18)	C27—C28—C29—C30	−0.6 (3)
C2—C3—C19—C18	−107.24 (17)	C28—C29—C30—C25	0.7 (3)
C3—C4—S1—C5	60.62 (15)	C30—C25—C26—C27	−0.8 (3)
C4—C3—C18—C19	−108.84 (18)	C31—C32—C33—C34	−0.7 (4)
C4—C3—C19—C18	105.47 (18)	C32—C31—C36—C35	0.2 (3)
C5—C6—C7—C8	−176.77 (16)	C32—C31—N2—C20	−68.0 (2)
C5—C6—C11—C10	176.14 (19)	C32—C31—N2—C24	99.79 (19)
C6—C5—N1—C1	−67.81 (19)	C32—C33—C34—C35	1.7 (5)
C6—C5—N1—C12	123.27 (14)	C33—C34—C35—C36	−1.7 (5)
C6—C5—S1—C4	56.71 (12)	C34—C35—C36—C31	0.7 (4)
C6—C7—C8—C9	1.3 (3)	C36—C31—C32—C33	−0.2 (3)
C7—C6—C11—C10	1.2 (3)	C36—C31—N2—C20	112.91 (19)
C7—C8—C9—C10	0.1 (4)	C36—C31—N2—C24	−79.3 (2)
C8—C9—C10—C11	−0.8 (4)	C37—C22—C23—S2	77.21 (18)
C9—C10—C11—C6	0.1 (4)	C38—C22—C23—S2	145.15 (14)
C11—C6—C7—C8	−1.9 (3)	N1—C1—C2—C3	−73.44 (18)
C12—C13—C14—C15	0.5 (3)	N1—C5—C6—C7	−18.2 (2)
C13—C12—C17—C16	0.6 (3)	N1—C5—C6—C11	167.02 (15)
C13—C12—N1—C1	−62.6 (2)	N1—C5—S1—C4	−78.17 (12)
C13—C12—N1—C5	107.12 (17)	N1—C12—C13—C14	−179.08 (17)
C13—C14—C15—C16	0.9 (4)	N1—C12—C17—C16	178.48 (19)
C14—C15—C16—C17	−1.5 (4)	N2—C20—C21—C22	−72.77 (18)
C15—C16—C17—C12	0.8 (4)	N2—C24—C25—C26	−18.8 (2)
C17—C12—C13—C14	−1.2 (3)	N2—C24—C25—C30	166.66 (15)
C17—C12—N1—C1	119.57 (18)	N2—C24—S2—C23	−77.24 (12)
C17—C12—N1—C5	−70.7 (2)	N2—C31—C32—C33	−179.3 (2)
C18—C3—C4—S1	148.55 (15)	N2—C31—C36—C35	179.3 (2)
C19—C3—C4—S1	80.74 (19)	O1—C1—C2—C3	106.17 (17)
C20—C21—C22—C23	87.61 (17)	O1—C1—N1—C5	−176.34 (15)
C20—C21—C22—C37	−59.3 (2)	O1—C1—N1—C12	−7.7 (2)
C20—C21—C22—C38	−128.05 (16)	O2—C20—C21—C22	106.51 (17)
C21—C20—N2—C24	3.7 (2)	O2—C20—N2—C24	−175.58 (15)
C21—C20—N2—C31	170.18 (14)	O2—C20—N2—C31	−9.1 (2)
C21—C22—C23—S2	−69.90 (17)	S1—C5—C6—C7	−152.08 (13)
C21—C22—C37—C38	−107.87 (17)	S1—C5—C6—C11	33.10 (19)
C21—C22—C38—C37	107.97 (17)	S1—C5—N1—C1	67.73 (17)
C22—C23—S2—C24	62.03 (13)	S1—C5—N1—C12	−101.19 (13)
C23—C22—C37—C38	106.03 (17)	S2—C24—C25—C26	−152.98 (13)
C23—C22—C38—C37	−108.16 (17)	S2—C24—C25—C30	32.50 (19)
C24—C25—C26—C27	−175.41 (16)	S2—C24—N2—C20	66.84 (18)
C24—C25—C30—C29	174.70 (17)	S2—C24—N2—C31	−99.88 (14)

### Hydrogen-bond geometry (Å, º)

**Table:**

*D*—H···*A*	*D*—H	H···*A*	*D*···*A*	*D*—H···*A*
C18—H18*A*···O2^i^	0.97	2.60	3.364 (2)	136
C38—H38*A*···O1	0.97	2.53	3.384 (2)	146

Symmetry code: (i) *x*, *y*−1, *z*.
